# The association between the route of nutrition and serum levels of adipokines in critical illness: a pilot study

**DOI:** 10.3906/sag-1911-165

**Published:** 2020-06-23

**Authors:** Kürşat GÜNDOĞAN, Ender DOĞAN, Nurhayat Tuğra ÖZER, Gülşah GÜNEŞ ŞAHİN, Serap ŞAHİN, Murat SUNGUR, İsmail Hakkı AKBUDAK, Sebahattin MUHTAROĞLU, Muhammet GÜVEN, Thomas R. ZIEGLER

**Affiliations:** 1 Division of Medical Intensive Care, Department of Medicine, School of Medicine, Erciyes University, Kayseri Turkey; 2 Department of Medicine, School of Medicine, Erciyes University, Kayseri Turkey; 3 Department of Clinical Nutrition, Institute of Health Sciences, Erciyes University, Kayseri Turkey; 4 Department of Biochemistry, School of Medicine, Erciyes University, Kayseri Turkey; 5 Nutrition and Metabolic Support Service, Emory University Hospital, Atlanta, Georgia USA; 6 Division of Endocrinology, Metabolism and Lipids, Department of Medicine, School of Medicine,Emory University, Atlanta, Georgia USA

**Keywords:** Adipokine, critical illness, enteral nutrition, parenteral nutrition.

## Abstract

**Background/aim:**

Adipokines play an important role in the regulation of metabolism. In critical illness, they alter serum levels and are suspected to worsen clinical outcomes. But the effect of the route of nutrition on adipokines is not known. The purpose of this study was to evaluate the association between the route of nutrition and adipokine levels in critically ill patients.

**Materials and methods:**

This prospective study was performed in an intensive care unit (ICU). Patients admitted to the ICU for least 72 h and receiving either enteral nutrition (EN) via tube feeding or parenteral nutrition (PN) were enrolled. Serum was obtained at baseline, 24 h, and 72 h for concentrations of leptin, adiponectin, resistin, glucagon–like peptide 1 (GLP–1), insulin–like growth factors 1 (IGF–1), and ghrelin.

**Results:**

A total of 26 patients were included in the study. Thirteen patients received EN and 13 patients received PN. In the PN group, leptin level significantly increased (P = 0.037), adiponectin and ghrelin significantly decreased during follow up (P = 0.037, P = 0.008, respectively). There was no significant change between all adipokines in the EN group and resistin, IGF–1 and GLP–1 in the PN group during follow up. Resistin levels were markedly lower in the EN group at both 24 h (P = 0.015) and 72 h (P = 0.006) while GLP–1 levels were higher in the EN group at baseline, 24 h, and 72 h (P = 0.018, P = 0.005, and P = 0.003, respectively). There were no differences in leptin, adiponectin, IGF–1, and ghrelin levels over time.

**Conclusion:**

The delivery of EN in critical illness was associated with decreased resistin levels and increased GLP–1 levels. Thus, the route of nutrition may impact the clinical outcome in critical illness due to adipokines.

## 1. Introduction

Adipose tissue, a dynamic endocrine organ, is composed of adipokines, which have a number of biologically active proteins [1]. Adipokines play a significant role in the regulation of appetite and satiety, energy expenditure, fat distribution, insulin sensitivity and secretion, inflammation and acute-phase responses, immunity, blood pressure, homeostasis, and endothelial functions [2]. Many adipokines have been identified, and leptin, adiponectin, resistin, glucagon–like peptide 1 (GLP–1), insulin–like growth factors 1 (IGF–1), and ghrelin are the most closely studied [3]. 

Adipose tissue might have an important role in the adaptation to critical illness, which is a multifactorial heterogeneous disease accompanied by inflammation and insulin resistance. It changes the secretory function and leads to major changes in adipokine levels [4]. Therefore, adipokines are suspected of affecting clinical outcomes in critical illness [3,5,6]. At present, the effect of some adipokines in critical illness is debated but many studies have shown that adipokines may cause metabolic alterations depending on changes in morphological, physiological, and metabolic functions in adipose tissue due to critical illness [7–12].

When oral intake is not possible and the gastrointestinal system is functional in critically ill patients, EN should be preferred. If oral and EN are contraindicated, PN is performed in critically ill patients [13]. EN prevents intestinal villus atrophy, protects against ischemic reperfusion injury by stimulating intestinal perfusion, reduces bacterial permeability, and prevents the development of systemic infection and multi-organ failure by protecting intestinal barriers in critically ill patients [14]. EN affects direct intestinal adipose tissue especially related to the incretin effect, which is responsible for adipokines such as GLP–1 and glucose–dependent insulin tropic peptide (GIP) [15,16]. Hence, adipokines are thought to be a pathway for the therapeutic benefits of EN [17]. In a study conducted on patients undergoing ileum resection due to intestinal injury in Crohn’s disease, it was reported that EN contributed adipocyte morphology restoration and reduced inflammation in mesenteric fat tissue [18].

The effects of EN and PN on circulating adipokines have not been studied well in critically ill patients. The aim of this pilot study was to investigate the effects of EN and PN on these hormones in critically ill patients. 

## 2. Materials and methods

The present study was performed prospectively in the Medical and Surgical ICU. This study was approved by the local Ethics Committee and all patients provided informed consent. This study was a prospective, observational, and single–centered pilot study with secondary analysis of the association between refeeding hypophosphatemia and serum appetite-regulating hormone levels in critically ill patients.

Patients aged ≥ 18 years and admitted to the ICU for at least 72 h and who received nutrition support via EN or PN were included in this study. Patients with hypophosphatemia (serum phosphorus level ≤ 2.4 mg/L), chronic renal failure, diabetic ketoacidosis, or hyperparathyroidism at the onset of nutrition, undergoing treatment for chronic liver disease or biliary tract diseases (except cholecystectomy), or gastric bypass surgery were excluded from the study.

Patient demographic characteristics, the reason for ICU admission, APACHE–II score, SOFA score, Charlson comorbidities score, and Nutrition Risk in the Critically İll Score (NUTRIC score) were recorded upon ICU admission. IR was evaluated by using the HOMA model [HOMA-IR = fasting insulin (µIU / mL) × fasting glucose (mmol/L)/ 22.5] [19]. Additionally, the route of nutrition, daily calorie intake and content and time to feeding of patients were recorded. The PN group accepted patients who received PN and the EN group accepted patients who received EN.

Blood samples were collected in order to measure serum leptin, resistin, adiponectin, GLP–1, IGF–1 and ghrelin at baseline, 24 h, and 72 h. Blood samples were processed with Trasylol, an inhibitor of pancreatic trypsin in order to prevent degradation of peptide hormones by pancreatic trypsin. 

Nutrition initiation time and type, target calorie requirement, and, enteral/parenteral product selection were set according to ESPEN guidelines after ICU admission [20]. Patients were given 20–25 kcal/kg/d energy in acute phase of illness, and 25–30 kcal / kg/d energy in chronic phase of the illness. Each patient received only EN or only PN.

Patients with normal gastrointestinal function received EN. Nutrition therapy was started with 10 mL/h. The nutritional goal was reached by increasing by 10 mL/4 h. Patients were routinely given standard enteral formula. Some patients were given a high–fat, low–carbohydrate enteral formula, a low–volume, high–energy enteral formula, and a diabetes specific formula for treatment.

PN was given if EN was contraindicated due to disturbed gastrointestinal function. For PN, standard commercial products were used. Compounder TPN was prepared to different content–carbohydrates (50–60%), protein (15–20%), fat (20–30%) of calories all in one bag in our hospital.

Serum adipokines were analyzed at 3 time points (baseline, 24 h, and 72 h) by enzyme–linked immunosorbent assay (ELISA) for leptin (DiaSource, Belgium), adiponectin (Biovendor, Czech Republic), resistin (Biovance Technologies, USA), GLP–1 (Ray Biotech, USA), and ghrelin (Phoenix, USA). IGF–1 was analyzed with the autoanalyzer in the hormone laboratory.

### 2.1. Statistical analysis

Statistical analysis was conducted using IBM SPSS Statistics 22 (IBM Corp., Armonk, NY, USA). Categorical data are presented as n (%), continuous data as median (interquartile range [IQR]). Categorical data were compared between 2 groups using the chi-square exact test. Continuous data were compared between 2 groups using Mann Whitney U test. Friedman test was used to determine the serum adipokines levels period. Differences were considered statistically significant at P > 0.05.

## 3. Results

A total of 26 patients were included in this study. There were nine (35%) females and 17 (65%) males. The median age was 69 (53–75) years. Patient demographic data are presented in Table 1. The median APACHE-II score was 24 (17–28) and the median SOFA score was 4.0 (0–13.0) at admission to ICU (Table 1).

**Table 1:** Patient demographic and clinical characteristics.

Variables	Total(n = 26)	EN group(n = 13)	PN group(n = 13)	P
Age (year), median (IQR)	69 (53–75)	69 (44–76)	68 (56–75)	0.685
Sex, n (%) Male Female	17 (65) 9 (35)	9 (64) 5 (36)	8 (67) 4 (33)	1
Body Mass Index (kg/m2), median (IQR)	27 (22–33)	26 (23–28)	27 (22–33)	0.153
ICU admission type, n (%) Medical Surgery	17 (65) 9 (35)	10 (71) 4 (29)	7 (58) 5 (42)	0.986
Reason for ICU admission, n (%) Malignancy Sepsis Neurological disorders Trauma Intoxication Respiratory failure Gastrointestinal bleeding	9 (35) 5 (19) 5 (19) 3 (12) 2 (7) 1 (4) 1 (4)	2 (15) 2 (15) 5 (39) 2 (15) 1 (8) 1 (8) 0	7 (54) 3 (22) 0 1 (8) 1 (8) 0 1 (8)	0.043
Insulin resistance, n (%)	13 (50)	5 (39)	8 (62)	0.434
Charlson comorbidity index, median (IQR)	3 (0–8)	2 (0–8)	4 (0–8)	0.287
APACHE II score, median (IQR)	24 (17–28)	22 (17–28)	25 (18–28)	0.633
SOFA score, median (IQR)	4.0 (0–13.0)	3.0 (0–13.0)	4 (0–13.0)	0.880
NUTRIC score, median (IQR)	4 (3–5)	5 (3–5)	4 (3–5)	0.011
Length of hospital stay (day), median (IQR)	31 (8–150)	34 (8–125)	28 (9–150)	0.169
Length of ICU stay (day), median (IQR)	20 (2–125)	27 (2–125)	13 (2–45)	0.179
Mortality, n (%)	14 (54)	9 (69)	5 (38)	0.238

APACHE II, Acute Physiology and Chronic Health Evaluation; SOFA, Sequential Organ Failure Assessment, Nutrition Risk Screening–2002.

A total of 13 patients (50%) received EN via feeding tube and 13 patients (50%) received PN during the 3–day period of observation. In the EN group, nutrition was temporarily interrupted in 3 patients due to high gastric residual volumes and in 1 patient due to tracheostomy. The target calorie requirement, daily energy intake, and macronutrient content (the composition of carbohydrate, protein, and lipid in the EN group; the composition of glucose, amino acid, and lipid in the PN group) of the patients are shown in Table 2.

**Table 2:** Time to feeding, target calorie requirement, daily calorie intake, percentage energy intake/ requirement, daily carbohydrate/glucose, total protein/amino acid, and lipid delivered for the EN and PN groups.

Variables	Total (n = 26)	EN group (n = 13)	PN group (n = 13)	P
Time to feeding (h), median (IQR)	24 (20–48)	24 (17–35)	26 (23–62)	0.155
Target calorie requirement (kcal), median (IQR)	1600 (1400–1856)	1750 (1365–1937)	1600 (1400–1800)	0.515
EN product, n (%) Standard enteral formula High–fat, low–carbohydrate enteral formula Low–volume, high–energy enteral formula Diabetes specific formula PN product TPN (with compounder) TPN (commercial PN products)		6 (46.2) 4 (30.8) 1 (11.0) 1 (11.0) 1 (11.0)	11 (84.6) 2 (14.4)	
Daily calorie intake (kcal), median (IQR) Day 1 Day 2 Day 3	1188 (1053–1375) 1592 (1272–1856) 1474 (1200–1811)	1105 (946– 1323) 1750 (1296–1911) 1474 (1061–1885)	1275 (1122–1386) 1584 (1200–1700) 1600 (1200–1700)	0.219 0.799 0.676
Percentage energy intake / requirement Day 1 Day 2 Day 3	74.8 (67.6–86.6) 100.0 (98.0–100.0) 100.0 (90.0–100.0)	74.6 (54.4–81.6) 99.4 (96.8–100) 98.2 (77.4–100)	83.9 (68.4–100) 99.5 (99.0–100) 100 (100–100)	0.173 0.190 0.058
Daily carbohydrate/glucose delivered (g/d), median (IQR) Day 1 Day 2 Day 3	137.5 (96.8–163.6) 162.4 (133.6–200.0) 142.2 (81.6–200.0)	106.5 (77.7–130.5) 142.2 (106.6–198.0) 90.7 (56.6–140.4)	159.7 (138.9–170.6) 186.5 (152.5–200.0) 187.5 (152.5–200.0)	0.002 0.057 0.001
Daily protein/amino acid delivered (g/d), median (IQR) Day 1 Day 2 Day 3	55.0 (45.8–67.6) 72.5 (55.1–87.4) 72.4 (51.8–81.0)	48.7 (44.3–65.1) 72.5 (53.9–89.6) 72.4 (47.2–85.3)	61.9 (53.4–68.2) 74.6 (57.1–80.0) 75.0 (52.5–80.0)	0.1750.9770.552
Daily lipid delivered (g/d), median (IQR) Day 1 Day 2 Day 3	44.5 (37.6–47.5) 53.3 (45.7–71.5) 53.3 (40.0–68.0)	45.1 (38.3–62.6) 70.6 (52.0–93.4) 56.4 (41.4–93.4)	41.3 (37.0–45.5) 49.7 (40.0–53.3) 50.0 (40.0–53.3)	0.269 0.038 0.177

In the PN group, the leptin level significantly increased (P = 0.037); adiponectin and ghrelin significantly decreased during follow up (P = 0.037, P = 0.008, respectively). There was no significant change between all adipokines in the EN group and resistin, IGF–1 and GLP–1 in the PN group during follow up (Table 3).

**Table 3:** Serum adipokines levels at baseline, 24 h, and 72 h to EN group and PN group.

	EN group (n = 13)				PN group (n = 13)			
	Baseline	24 h	72 h	P	Baseline	24 h	72 h	P
Leptin (ng / mL), median (IQR)	2.0 (1.3–6.7)	2.5 (1.8–5.6)	3.8 (2.1–8.9)	0.092	1.3 (0.7–2.4)	2.3 (0.6–4.1)	2.3 (1.3–3.9)	0.037
Adiponectin (ng / mL), median (IQR)	25.2 (15.0–37.9)	20.2 (16.6–33.2)	28.6 (19.9–53.2)	0.368	32.9 (23.2–44.4)	25.9 (15.1–31.1)	21.7 (15.4–33.1)	0.037
Resistin (ng /mL), median (IQR)	905.6 (589.8–1393.3)	594.9 (354.7–861.8)	693.6 (464.5–931.7)	0.063	1373.3 (973.0–3332.3)	1615.2 (808.5–2532.9)	1543.4 (882.5–2183.6)	0.794
IGF–1 (ng/ mL), median (IQR)	85.6 (65.2–96.7)	43.9 (28.2–73.5)	55.3 (28.0–78.5)	0.098	52.7 (29.3– 88.1)	40.7 (27.3–74.0)	32.2 (25.0–66.6)	0.913
GLP–1 (pg/ mL), median (IQR)	3.1 (2.3–5.5)	5.0 (2.5–13.1)	4.8 (2.3–39.1)	0.232	0.6 (0.4–2.8)	0.9 (0.5–3.5)	0.7 (0.5–3.2)	0.662
Ghrelin (ng /mL), median (IQR)	1.1 (0.9–1.3)	1.0 (0.9–1.3)	1.2 (1.0–1.6)	0.500	1.2 (1.0–1.4)	1.2 (1.1–1.4)	1.0 (0.9–1.2)	0.008

Serial serum adipokine levels in the EN and PN groups are presented in Figure. There was no difference between the EN and PN groups for serum leptin, adiponectin, IGF–1, and ghrelin levels at baseline, 24 h, or 72 h.

Serum resistin levels at 24 h and 72 h were significantly lower in the EN group compared to the PN group (P = 0.014, P = 0.005, respectively; Figure). However, there was no significant difference between the EN and PN groups in serum resistin levels at baseline. In contrast, serum GLP–1 levels in the EN group were significantly higher at baseline, 24 h, and 72 h compared to the PN group (P = 0.016, P = 0.004, P = 0.002, respectively; Figure).

**Figure:**
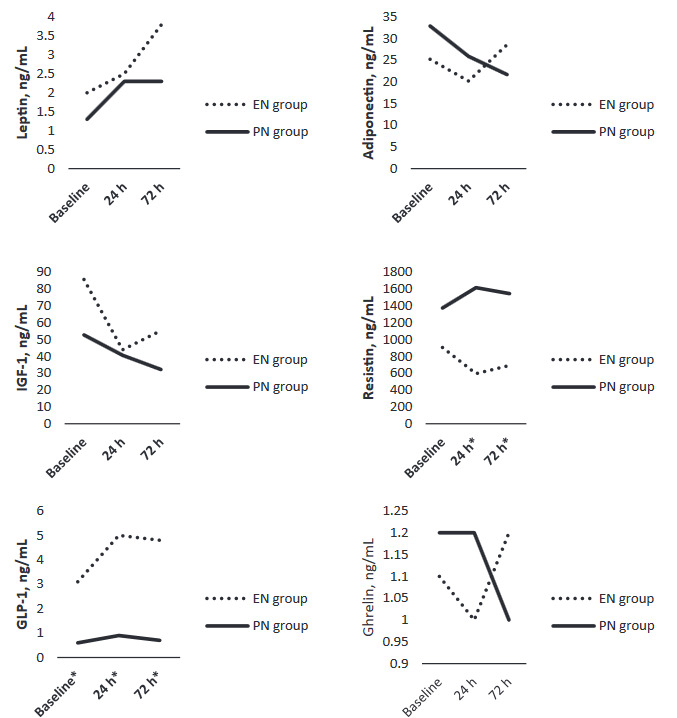
Serial serum adipokine levels in the EN and PN group. P < 0.05, Insulin -like growth factors 1 (IGF -1); GLP-1, Glucagon -like peptide 1 (GLP -1).

## 4. Discussion

This pilot study was conducted to investigate the relationship between serum adipokine levels and the route of nutrition in a mixed cohort of adult critically ill patients. We determined that the leptin level significantly increased, adiponectin and ghrelin significantly decreased during follow up in PN group, and there were decreased resistin levels as described in catabolic hormones and increased GLP–1 levels as described in anabolic hormones in the EN group.

Leptin levels are usually increased due to the rise in endotoxins, certain cytokines, and glucocorticoids in critical illness, although studies of leptin levels are conflicting [4,21]. Leptin is also described as an indicator of fasting or malnutrition [22]. The level of leptin in critical illness is contradictory in the literature, leptin levels were increasing during follow-up in both groups. In a study, critical patients who were received continuous enteral nutrition were followed for 14 days. Similarly to our findings, the level of leptin increased during follow up, but was higher than our patients [23]. In our study, the PN group had higher malnutrition risk than the EN group according to the NUTRIC score. Consequently, we observed that leptin levels in the EN group were higher than the PN group for the three-time points even if statistically nonsignificant. 

In critical illness, glucose homeostasis is often impaired and insulin resistance is a common condition due to the presence of hyperglycemia and hyperinsulinemia. Lower adiponectin and leptin levels may contribute to insulin resistance [24]. While it was contradictory in the EN group, it decreased significantly during the follow-up in the PN group (P < 0.05) in our study. Also, insulin resistance was higher in the PN group. As expected, serum adiponectin and leptin levels in the PN group were relatively lower than in the EN group. Resistin is related to inflammation induced insulin resistance and is increased in critical illness. But, serum resistin levels was relatively decreasing in EN group, it was increasing in PN group and EN group were significantly lower than in the PN group (P < 0.05). We believe that these are due to the incretin effect associated with EN. McKenzie et al. evaluated adipokine levels in patients with acute pancreatitis and received EN during the first 72 h after hospital admission. Similar to our study, leptin and adiponectin levels increased, while the level of resistin decreased [17].

GLP–1, 1 of the 2 known incretins, is known to have increased release with EN. Its levels increased in both group during the follow-up and were determined higher in the EN group (P < 0.05). Similar to our study, several previous studies have also reported elevated GLP–1 in patients who received EN in critical illness [16,25]. This data may provide evidence based on nonnutritional effects of EN and superiority to PN with the incretin effect.

IGF–1 is a sensitive indicator of nutritional condition and inflammation and is decreased in cases of insufficient nutrition and presence of critical illness. In the present study, serum IGF–1 levels decreased in both groups and were lower in the EN group than in the PN group, significantly. We considered that this was associated with the daily calorie intake. The percentage energy intake requirement in the PN group was higher than in the EN group in our study because EN was frequently interrupted for various reasons (nutrition intolerance, diagnosis, treatment interventions). Therefore, it is easier to reach the target calorie requirement with PN [16,26]. Similarly, Isley et al. observed in a study of 15 patients that IGF–1 levels increased with sufficient energy and protein support. They reported a temporary decrease followed by an increase in IGF–1 levels after administering low protein content nutrition with normal caloric levels. They observed a decrease in IGF–1 levels in cases of insufficient nutrition [27]. 

There was an increase in ghrelin levels in the EN group and, significantly, a decrease in the PN group with an extended duration of nutrition. Both groups had similar serum ghrelin levels at baseline. Similar to our study, in a study conducted in critical patients fed enterally, the level of ghrelin increased in a similar follow up period. However, the level of ghrelin was lower in our patients [23]. Because the increase in the EN group might be associated with the release of ghrelin by gastric oxyntic cells. Enteral nutrition might therefore, trigger ghrelin secretion more effectively than PN. Similar to our study, Hagiwara et al. conducted a study with 4 groups of 15 rodents each, who received total enteral nutrition conventional (TEN - C), total enteral nutrition immunonutrition (TEN-I), total parenteral nutrition (TPN), and saline. The daily calorie intake of all groups was approximately similar. The study observed that ghrelin levels were lower in PN compared to EN [28].

Although the role of adipokines in critical illness is not understood clearly, significant alterations of circulating adipokines may be associated with poor clinical outcomes in critically ill patients. Even though conclusive evidence that leptin and adiponectin lead to poor clinical outcomes (increased mortality, inflammation, and development of multiple organ dysfunction syndromes) is not provided, some studies present the existence of this relationship [29,30]. In all studies of resistin, increased levels of blood resistin are powerfully associated with severe inflammation and increased risks of organ failure and mortality [31–33]. High ghrelin levels are accepted as a positive predictor of ICU survival and decreased ghrelin levels can lead to inflammation and length of mechanical ventilation stay [4]. Clinical outcomes due to adipokine levels may differ in artificial nutrition (EN or PN). Although most of our data were not statistically significant, it was shown that EN increases anabolic hormones and reduces catabolic hormones in critical illness. In addition, the delivery of EN was associated with a lower level of hormones that cause insulin resistance.

This study had several limitations, including the single–center design, a relatively small number of patients, heterogeneous study groups, and a lack of follow up of clinical outcome (mechanical ventilation stay, inflammation, etc.). However, its strengths were the comprehensive panel of adipokines and the first look, to our knowledge, at the route of nutrition in these hormonal responses in ICU patients.

In conclusion, this study indicates that EN may help to correct abnormal processes in critically ill patients by decreasing resistin levels and increasing GLP–1 levels. Adipokines may be associated with poor clinical outcomes of critically ill patients, including higher inflammation, greater risk of organ dysfunction, and mortality. The importance of nutrition for normal adipokine levels in critically ill patients is an indisputable fact. At this point, the route of nutrition can be an important key. We conclude that the nonnutritional effects of EN and its relationship with the incretin effect should not be ignored. Therefore, the relationship between adipokines and nutrition should be clarified. Further studies with a larger number of patients are necessary to choose the nutrition type that can lead to the least complications.

## Acknowledgements

This study was supported by Erciyes University Scientific Research Unit (TSA-2016-6082).

## Conflict of Interest 

The authors declare that there is no conflict of interest.
